# Effects of Long-Term Serum Starvation on Autophagy, Metabolism, and Differentiation of Porcine Skeletal Muscle Satellite Cells

**DOI:** 10.3390/vetsci12010011

**Published:** 2024-12-30

**Authors:** Yi Wang, Juan Gao, Bojun Fan, Yuemin Hu, Yuefei Yang, Yajie Wu, Jiaqiao Zhu, Junwei Li, Feng Li, Huiming Ju

**Affiliations:** 1 College of Veterinary Medicine, Yangzhou University/Jiangsu Co-Innovation Center for Prevention and Control of Important Animal Infectious Diseases and Zoonosis, Yangzhou 225009, China; wyi@yzu.edu.cn (Y.W.); mz120201425@stu.yzu.edu.cn (J.G.); mx120211006@stu.yzu.edu.cn (B.F.); mx120190778@stu.yzu.edu.cn (Y.H.); yfyang@yzu.edu.cn (Y.Y.); mx120211004@stu.yzu.edu.cn (Y.W.); jqzhu1998@163.com (J.Z.); lijunwei@yzu.edu.cn (J.L.); 2 Department of Reproductive Medicine Center, Northern Jiangsu People’s Hospital Affiliated to Yangzhou University/Clinical Medical College, Yangzhou University, Yangzhou 225009, China; minprince@163.com

**Keywords:** serum starvation, skeletal muscle satellite cell, autophagy, cell metabolism, differentiation

## Abstract

This study investigated the effects of long-term serum starvation on autophagy, metabolism, and differentiation of porcine skeletal muscle satellite cells (SMSCs) and elucidated the role of autophagy in skeletal muscle development. Our findings provide a theoretical basis for improving meat production in domestic pigs. The SMSCs isolated and preserved in our laboratory were revived and divided into six groups based on the culture medium serum concentration to simulate varying levels of serum starvation: 20% serum (control group), 15% serum (mild serum starvation group), 5% serum (severe serum starvation group), and their autophagy inhibition groups supplemented with 3-methyladenine. After 96 h of culture, the apoptosis rate, mitochondrial membrane potential, reactive oxygen species, and ATP were measured to evaluate the effects of serum starvation on the SMSCs’ metabolism. Additionally, the levels of autophagy-related proteins, autophagosomes, and autolysosomes were measured to investigate the impact of long-term serum starvation on autophagy. The expression of proteins associated with myogenic and adipogenic differentiation (MHC, MyoD1, peroxisome proliferator-activated receptor *γ*, and lipoprotein lipase) as well as lipid content were also determined to investigate the effects of long-term serum starvation on SMSC differentiation. The results showed that long-term serum starvation induced autophagy through the AMPK/mTOR signaling pathway, accelerated cell metabolism and apoptosis, exacerbated reactive oxygen species accumulation, and inhibited myogenic and adipogenic differentiation of SMSCs. Moreover, these effects were positively correlated with the level of serum starvation. In addition, serum starvation-induced autophagy moderately promoted the myogenic and adipogenic differentiation of SMSCs; however, these effects were insufficient to counteract the inhibition of cell differentiation by long-term serum starvation. This study provides insight into leveraging serum starvation as a stressor to regulate muscle growth and metabolism in domestic pigs.

## Simple Summary

This study investigated the effects of long-term serum starvation on autophagy, metabolism, and differentiation of porcine skeletal muscle satellite cells (SMSCs) and elucidated the role of autophagy in skeletal muscle development to provide a theoretical basis for improving meat production in domestic pigs. The results showed that long-term serum starvation induced autophagy through the AMPK/mTOR signaling pathway, accelerated cell metabolism and apoptosis, exacerbated reactive oxygen species accumulation, and inhibited myogenic and adipogenic differentiation of SMSCs. In addition, serum starvation-induced autophagy moderately promoted the myogenic and adipogenic differentiation of SMSCs. However, these effects were insufficient to counteract the inhibition of cell differentiation by long-term serum starvation. This study provides insight into leveraging serum starvation as a stressor to regulate muscle growth and metabolism in domestic pigs.

## 1. Introduction

Skeletal muscle satellite cells (SMSCs), which were discovered by Mauro in 1961, are undifferentiated and quiescent myogenic precursor cells located between the basal lamina and sarcolemma of adult muscle tissue [1]. When muscle growth is impaired or stressed by degenerative muscular diseases, SMSCs can play a crucial role in muscle development, maintenance, and regeneration by forming myofibers through myogenic differentiation. These cells can also directly influence the growth of porcine skeletal muscle and meat production [2,3,4]. Autophagy involves the formation of autophagosomes, which facilitate the recycling of biological macromolecules such as proteins and organelles like mitochondria into the lysosome [5]. Within the lysosome, these components are degraded into small molecules such as amino acids and monosaccharides, enabling the reuse of energy and materials. Autophagy has recently received great attention because of its role in skeletal muscle metabolic homeostasis and disease progression. Skeletal muscle differentiation is a stress-related process, and the growth and differentiation of skeletal muscle can be promoted by activation of the AMPK signaling pathway, caspase activation, excessive reactive oxygen species (ROS) accumulation, mitochondrial fission, and changes in autophagic flux [6]. Studies have shown that autophagic flux increases in skeletal muscle during hypoxia, disuse atrophy, starvation, and lack of exercise [7,8,9]. We previously confirmed that short-term (24 h) and mild (15% serum concentration) serum starvation promoted SMSC metabolism, induced autophagy via the AMPK/mTOR signaling pathway, and promoted myogenic and adipogenic differentiation in SMSCs [10]. We hypothesized that serum starvation plays an important role in the growth and metabolism of animal skeletal muscle. To further investigate the effects of serum starvation on porcine SMSCs, we induced long-term mild and severe serum starvation in SMSCs by reducing serum concentration for an extended period (96 h) and then evaluated the effects on the autophagy, metabolism, and differentiation of SMSCs. This study contributes to a comprehensive understanding of the effects of serum starvation on skeletal muscle growth and development, and the results lay a theoretical foundation for optimizing the use of serum starvation-induced autophagy to improve feed efficiency in animal production.

## 2. Materials and Methods

### 2.1. Cell Grouping and Treatment

Porcine SMSC cell lines were preserved in our laboratory. We resuscitated the cells and divided them into six groups when they reached 70–80% confluence. The 20% S group (the control group), the 15% S group, and the 5% S group refer to the cells grown in culture media containing 20%, 15%, and 5% serum (FBS, Ref#10270-106, Gibco, Emeryville, CA, USA). We subjected the above groups to autophagy inhibition via treatment with 5 mM of the autophagy inhibitor 3-methyladenine (3-MA, HY-19312, MCE, Plymouth, MN, USA) and named them 20% S + 3-MA group, 15% S + 3-MA group, and 5% S + 3-MA group, respectively. Autophagy-related proteins, cell metabolic indices, and differentiation abilities were detected after 96 h of culture under the corresponding conditions.

### 2.2. Detection of Cell Metabolism

The apoptosis detection kit (C1062S, Beyotime, Shanghai, China), ROS detection kit (S0033S, Beyotime, Shanghai, China), and MMP detection kit (C2006, Beyotime, Shanghai, China) were used to detect cell apoptosis rate, mitochondrial active oxygen (ROS) level, and mitochondrial membrane potential (MMP) level, respectively, by flow cytometry (Beckman Coulter, Fullerton, CA, USA). The concentration of ATP was determined by the ATP detection kit (S0026, Beyotime, Shanghai, China). The specific methods were similar to those in reference [10].

### 2.3. Detection of Autophagy

#### 2.3.1. Detection of Lysosomes and Autophagosomes

Mitochondria and lysosomes were stained with Mito-Tracker (C1048, Beyotime, Shanghai, China) and Lyso-Tracker (C1046, Beyotime, Shanghai, China); then, the cells were photographed by fluorescence microscope, and the fluorescence intensity was analyzed by ImageJ software 1.8.0_172 (NIH, Bethesda, MD, USA).

Lipofectamine™ 3000 (L3000150, Thermo, Waltham, MA, USA) diluted in Opti-MEM™ (31985070, Thermo, Waltham, MA, USA) was combined with premixed LC3B plasmid (842-pmCherry-EGFP-LC3B, Beyotime, Shanghai, China) solution at a 1:1 ratio for autophagosome detection. After incubation with DNA–lipid complex for 24 h, samples were observed under a fluorescence microscope.

#### 2.3.2. Detection of Autophagy-Related Protein Expression by Western Blot

Total protein was extracted from the cells, and tubulin (11224-1-AP, Proteintech, Rosemont, IL, USA) was used as the internal reference protein. The ratios of phospho-mTOR/mTOR (67778-1-lg and 66888-1-lg, Proteintech, Rosemont, IL, USA), phospho-AMPK/AMPK (GTX03702, GeneTex, Irvine, CA, USA and 66536-1-lg, Proteintech, Rosemont, IL, USA), LC3B-II/LC3B-I (ab229327, Abcam, Waltham, MA, USA) and p62/Tubulin (ab233207, Abcam, Waltham, MA, USA) were detected by Western blot. The gray values of hybrid bands were measured by ImageJ software 1.8.0_172 (NIH, Bethesda, MD, USA). The expression of the proteins was quantified and calculated as reported by [10,11].

### 2.4. Detection of Cell Differentiation

#### 2.4.1. Myogenesis and Lipogenesis Induction

Myogenesis differentiation medium and lipogenesis induction solution were used to induce myogenic and lipogenesis differentiation of SMSCs, respectively. The specific induction methods were similar to those in reference [10].

#### 2.4.2. Detection of Myogenic Differentiation Markers by Western Blot

MHC and MyoD1 antibodies (sc-53088 and sc-32758, Santa Cruz, TX, USA) were used to detect myogenesis. The specific methods used to detect myogenic differentiation markers are described in Section 2.3.2.

#### 2.4.3. Detection of Myogenic Differentiation Markers by Cellular Immunofluorescence

MHC, MyoD1 antibodies, and the FITC fluorescent secondary antibody (SA00003-1, Proteintech, Rosemont, IL, USA) were incubated after the SMSCs were fixed and permeabilized. DAPI was used to stain the cell nucleus, and laser confocal microscope (TCS SP8 STED, Leica, Wetzlar, Germany) was used to detect MHC and MyoD1 protein expression. ImageJ software 1.8.0_172 (NIH, Bethesda, MD, USA) was used to analyze the fluorescence intensity. Myogenic differentiation markers were detected as previously described [12].

#### 2.4.4. Detection of Adipogenic Differentiation Markers by qRT-PCR

After lipogenesis induction, we isolated total mRNA from the cells and reverse transcribed them into cDNA. We detected the expression of PPARγ and LPL mRNA by qRT-PCR with GAPDH gene as the internal reference and operated the reaction systems according to TB Green^®^ Premix Ex Taq™ II (RR82LR, Takara, Takatsuki, Japan). The related primers and the expression of the proteins was quantified as reported in [13].

#### 2.4.5. Detection of Fat Content with Oil Red O Staining

After lipogenesis induction, the cells were incubated with Oil Red O (G1262, Solarbio, Beijing, China) fixative, then stained with the Oil Red O staining solution. We observed the results under a microscope. Fat droplets were extracted with isopropanol and OD values at 510 nm were quantitatively detected.

#### 2.4.6. Detection of TG Levels

The supernatant of the cells after lipogenesis induction, digesting, and lysing was used as the test solution, and the absorbance value at 420 nm was detected by TG detection kit (BC0620, Solarbio, Beijing, China). The TG content of each group was quantitatively calculated according to the statistical cell number.

### 2.5. Statistical Analysis

All experiments were carried out with a minimum of three independent repeats. Data were presented as the x ± SD. Statistical differences between groups were analyzed using one-way ANOVA with GraphPad Prism7 software (GraphPad Software, San Diego, CA, USA), and differences were considered significant when *p* < 0.05.

## 3. Results and Analysis

The following data are presented in the order of the results from the 20% S, 20% S + 3-MA, 15% S, 15% S + 3-MA, 5% S, and 5% S + 3-MA groups.

### 3.1. Cell Metabolism

#### 3.1.1. Apoptosis Rate

**Figure 1:**
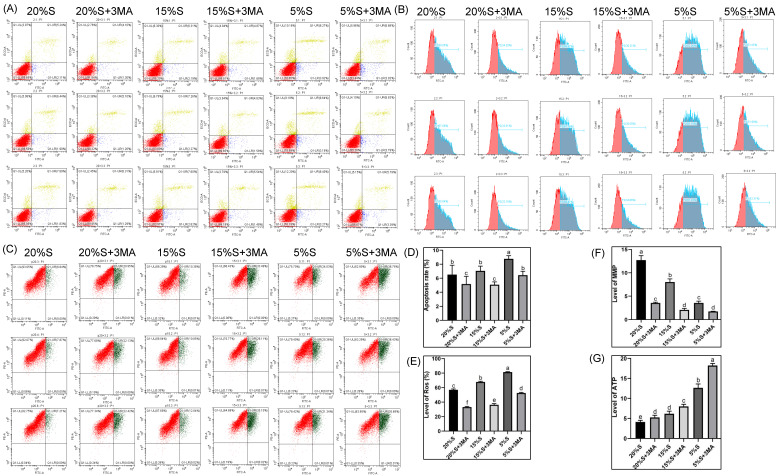
Detection and analysis of cellular metabolic levels. (**A**) Detection of apoptosis rate. Red spots represents the normal cells, yellow spots represents the late apoptotic cells and the mechanically damaged cells, and blue spots represents the early apoptotic cells. (**B**) Detection of ROS levels. (**C**) Detection of MMP. The red spots represents cells with high membrane potential, and the green ones represents cells with low membrane potential. (**D**) Analysis of apoptosis rate. (**E**) Analysis of ROS levels. (**F**) Analysis of MMP by JC-1 red-green fluorescence ratio. (**G**) Detection and analysis of intracellular ATP levels. The data are expressed as the mean ± SD (*n* = 3) of three experiments. The statistical significance of differences between groups was analyzed using one-way ANOVA, the same letters indicate the difference is not significant, while different letters indicate the difference is significant (*p* < 0.05).

The apoptosis rates in the six groups were 6.52 ± 1.33, 5.17 ± 1.10, 7.06 ± 0.67, 5.08 ± 0.40, 8.77 ± 0.45, and 6.43 ± 0.56, respectively, shown in Figure 1A,D. The apoptosis rates increased gradually in the 20% S, 15% S, and 5% S groups, and the apoptosis rate of 5% S was significantly higher than that of 20% S (*p* < 0.01). The protein expression levels were lower in groups with 3-MA than in the corresponding groups without 3-MA. These results indicate that serum starvation elevated the rate of apoptosis, and this effect was positively correlated with the level of serum starvation. Serum concentrations lower than those in the control group adversely affected cell metabolism. Additionally, 3-MA effectively mitigated the increase in apoptosis rates across all groups.

#### 3.1.2. ROS Level

The ROS levels in the six groups were 56.99 ± 1.11, 33.07 ± 1.1, 67.77 ± 0.9, 36.31 ± 1.7, 81.4 ± 0.53, and 52.55 ± 0.7, respectively, shown in Figure 1B,E. The ROS levels increased gradually in the 20% S, 15% S, and 5% S groups (*p* < 0.01). The protein expression levels were significantly lower in groups with 3-MA than in the corresponding groups without 3-MA (*p* < 0.01). The results suggest that increasing serum starvation was correlated with a gradual escalation in cellular oxidative stress and a significant increase in ROS levels. Moreover, the severity of cell damage was positively correlated with the level of serum starvation. Treatment with 3-MA effectively attenuated this increase in ROS levels.

#### 3.1.3. MMP Level

The MMP in each group was 12.72 ± 0.96, 3.57 ± 0.16, 8.02 ± 0.67, 2.08 ± 0.31, 3.57 ± 0.38, and 1.75 ± 0.04, respectively, shown in Figure 1C,F. The MMP decreased gradually in the 20% S, 15% S, and 5% S groups (*p* < 0.01). The protein expression levels were significantly lower in groups with 3-MA than in the corresponding groups without 3-MA (*p* < 0.01). Long-term nutritional deficiency hindered the SMSCs’ mitochondrial metabolism. Additionally, a significant decrease in MMP was observed upon inhibition of autophagy, suggesting that autophagy mitigated mitochondrial dysfunction.

#### 3.1.4. ATP Level

The ATP level in each group was 4.13 ± 0.41, 5.26 ± 0.54, 6.18 ± 0.62, 8.01 ± 0.52, 12.65 ± 0.97, and 18.22 ± 0.51, respectively, shown in Figure 1G. Compared with the 20% S group, the ATP levels in the remaining five groups gradually increased, suggesting that long-term serum starvation accelerated cell metabolism and induced compensatory production of ATP to compensate for the lack of cellular energy. However, the ATP levels further increased following autophagy inhibition, indicating that autophagy may play a positive role in maintaining normal cell metabolism by inhibiting intracellular energy consumption through specific pathways.

The results of Section 3.1 indicate that long-term serum starvation (mild and severe) showed inhibitory effects on cell growth and metabolism.

### 3.2. Autophagy

#### 3.2.1. Content of Autophagosomes and Lysosomes

**Figure 2:**
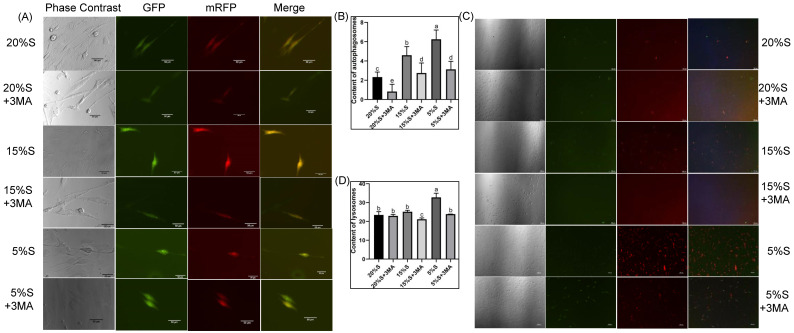
Detection and analysis of autophagosomes and lysosomes. (**A**) Detection of intracellular autophagic vesicles by fluorescence microscopy; (**B**) analysis of the average fluorescence value of Lyso-Tracker Red; (**C**) fluorescent probe localization of mitochondria (green) and lysosomes (red) in living cells; (**D**) analysis of the average fluorescence values of Lyso-Tracker Red. The data are expressed as the mean ± SD (*n*= 3) of three experiments. The statistical significance of differences between groups was analyzed using one-way ANOVA, the same letters indicate the difference is not significant, while different letters indicate the difference is significant (*p* < 0.05).

The number of autophagosomes in the six groups were 2.33 ± 0.52, 0.83 ± 0.75, 4.60 ± 0.89, 2.75 ± 1.04, 6.25 ± 0.96, and 3.13 ± 0.83, shown in Figure 2A,B. The fluorescence intensity of lysosomes in each group was 23.46 ± 1.72, 22.95 ± 0.75, 25.16 ± 0.74, 21.16 ± 0.68, 32.8 ± 2.18, and 23.87 ± 0.06, respectively, shown in Figure 2C,D. The fluorescence intensity of the lysosomes was significantly higher in the 5% S group than in the 20% S group (*p* < 0.05). Additionally, the 5% S + 3-MA group showed significantly reduced fluorescence (*p* < 0.05) compared with the 5% S group; however, fluorescence did not differ significantly among the other groups. The detection results of autophagosomes were basically consistent with those of lysosomes. These results indicate that increased serum starvation was correlated with an elevation in cellular autophagy, which was mitigated by 3-MA.

#### 3.2.2. Ratios of p-mTOR/mTOR and p-AMPK/AMPK 

**Figure 3:**
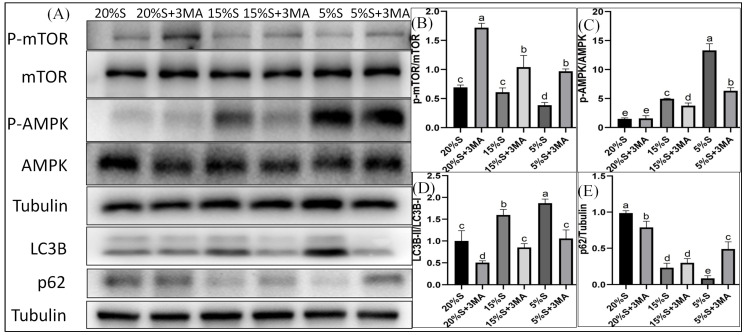
Detection and analysis of mTOR, p-mTOR, AMPK, p-AMPK, LC3B, and p62 expression by Western blot. (**A**) WB results of mTOR, p-mTOR, AMPK, p-AMPK, LC3B, and p62; (**B**) grayscale analysis of p-mTOR/mTOR; (**C**) grayscale analysis of p-AMPK/AMPK; (**D**) grayscale analysis of LC3B-II/LC3B-I; (**E**) grayscale analysis of p62. The data are expressed as the mean ± SD (*n* = 3) of three experiments. The statistical significance of differences between groups was analyzed using one-way ANOVA, the same letters indicate the difference is not significant, while different letters indicate the difference is significant (*p* < 0.05) (Supplementary File S1).

The gray value ratios of p-mTOR and mTOR proteins (p-mTOR/mTOR) related to the AMPK/mTOR signaling pathway in the six groups were 0.69 ± 0.04, 1.72 ± 0.07, 0.61 ± 0.07, 1.04 ± 0.20, 0.39 ± 0.05, and 0.97 ± 0.04, respectively, shown in Figure 3A,B. The ratios decreased gradually in the 20% S, 15% S, and 5% S groups (*p* < 0.01). The protein expression levels were significantly higher in groups with 3-MA than in the corresponding groups without 3-MA (*p* < 0.01). The gray value ratios of p-AMPK and AMPK proteins (p-AMPK/AMPK) in each group were 1.50 ± 0.17, 1.58 ± 0.46, 4.96 ± 0.08, 3.77 ± 0.47, 13.31 ± 1.15, and 6.33 ± 0.52, respectively, shown in Figure 3A,C. The ratios increased gradually in the 20% S, 15% S, and 5% S groups (*p* < 0.01). The protein expression levels were significantly lower in the 15% S and 5% S group with 3-MA than in the corresponding group without 3-MA (*p* < 0.01). The results suggest that serum starvation may induce autophagy through the AMPK/mTOR signaling pathway.

#### 3.2.3. Relative Expression of LC3B and p62 

The expression ratios of the autophagy marker proteins LC3B-II and LC3B-I (LC3B-II/LC3B-I) in the six groups were 1.00 ± 0.24, 0.50 ± 0.04, 1.60 ± 0.13, 0.85 ± 0.09, 1.87 ± 0.10, and 1.06 ± 0.20, respectively, shown in Figure 3A,D. The ratios increased gradually in the 20% S, 15% S, and 5% S groups. The ratios were significantly lower in groups with 3-MA than in the corresponding groups without 3-MA (*p* < 0.01). The relative expression of the p62 protein in each group was 0.99 ± 0.03, 0.79 ± 0.09, 0.23 ± 0.06, 0.30 ± 0.06, 0.09 ± 0.04, and 0.49 ± 0.10, respectively, shown in Figure 3A,E. The expression of the p62 protein decreased gradually in the 20% S, 15% S, and 5% S groups (*p* < 0.01). The expression levels in the 3-MA groups were higher than those in the corresponding groups without 3-MA. The results suggest that as the degree of serum starvation increases, the degree of cellular autophagy strengthens.

By measuring the indicators of Section 3.2, we observed that long-term serum starvation induced autophagy via the AMPK/mTOR pathway, with the level of autophagy showing a positive correlation with the severity of serum starvation.

### 3.3. Cell Differentiation

#### 3.3.1. Relative Expression of Myogenic Differentiation Markers

**Figure 4:**
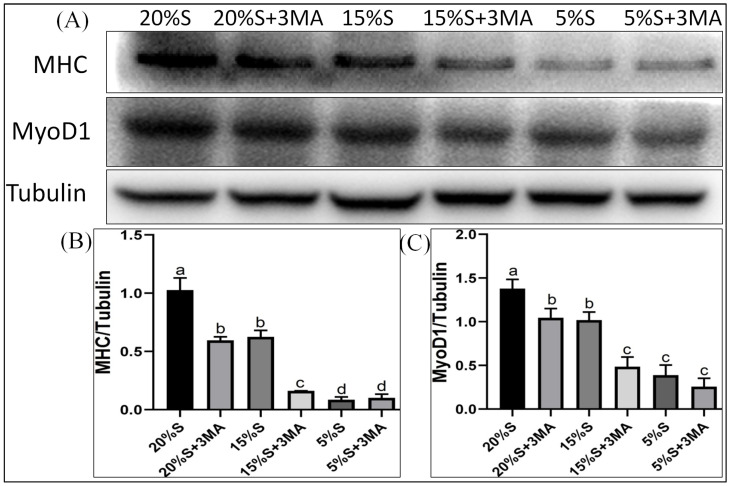
Detection and analysis of MHC and MyoD1 by Western blot. (**A**) WB results of MHC and MyoD1; (**B**) grayscale analysis of MHC; (**C**) grayscale analysis of MyoD1. The data are expressed as the mean ± SD (*n* = 3) of three experiments. The statistical significance of differences between groups was analyzed using one-way ANOVA, the same letters indicate the difference is not significant, while different letters indicate the difference is significant (*p* < 0.05) (Supplementary File S1).

The relative expression levels of MHC protein in each group were 1.03 ± 0.10, 0.60 ± 0.03, 0.63 ± 0.06, 0.16 ± 0.01, 0.09 ± 0.02, and 0.10 ± 0.03, respectively, shown in Figure 4A,B. The relative expression levels of MyoD1 protein in the six groups were 1.38 ± 0.11, 1.05 ± 0.11, 1.02 ± 0.09, 0.49 ± 0.11, 0.39 ± 0.12, and 0.26 ± 0.09, respectively, shown in Figure 4A,C. The expression of both proteins decreased gradually in the 20% S, 15% S, and 5% S groups (*p* < 0.01). The protein expressions were lower in groups with 3-MA than in the corresponding groups without 3-MA.

**Figure 5:**
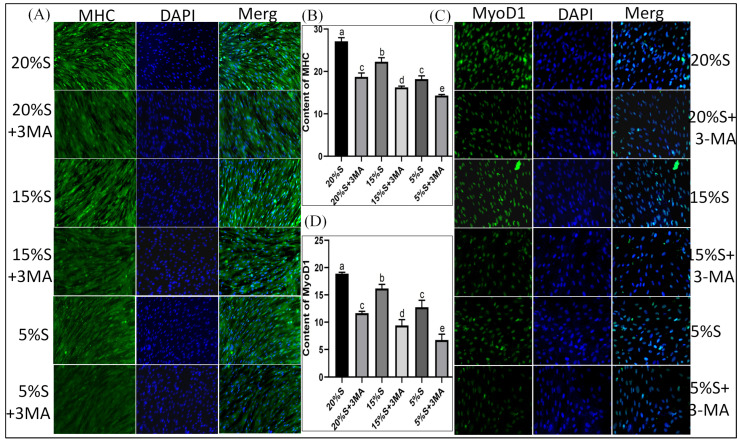
Detection and analysis of MHC and MyoD1 by cellular immunofluorescence. (**A**) Immunofluorescence staining results of MHC. The green part represents the positive cells that can express MHC, the blue part represents the nucleus; (**B**) quantitative analysis of MHC fluorescence; (**C**) immunofluorescence staining results of MyoD1. The green part represents the positive cells that can express MyoD1, the blue part represents the nucleus; (**D**) quantitative analysis of MyoD1 fluorescence. The data are expressed as the mean ± SD (*n* = 3) of three experiments. The statistical significance of differences between groups was analyzed using one-way ANOVA, the same letters indicate the difference is not significant, while different letters indicate the difference is significant (*p* < 0.05).

The fluorescence intensities of MHC protein in the six groups were 27.12 ± 0.83, 18.7 ± 0.93, 22.3 ± 0.95, 16.23 ± 0.32, 18.19 ± 0.78, and 14.31 ± 0.26, respectively, shown in Figure 5A,B. Those of MyoD1 protein in each group were 18.89 ± 0.25, 11.65 ± 0.34, 16.19 ± 0.74, 9.4 ± 1.07, 12.74 ± 1.27, and 6.74 ± 1.06, shown in Figure 5C,D. The expression of both proteins decreased gradually in the 20% S, 15% S, and 5% S groups (*p* < 0.01). The protein expression levels were lower in groups with 3-MA than in the corresponding groups without 3-MA (*p* < 0.01). The results were basically consistent with those of the Western blot analysis, which indicate that long-term serum starvation inhibited the myogenic differentiation of SMSCs, while autophagy mitigated the decline in myogenic differentiation during serum starvation.

#### 3.3.2. Relative Expression of Adipogenic Differentiation Markers

**Figure 6:**
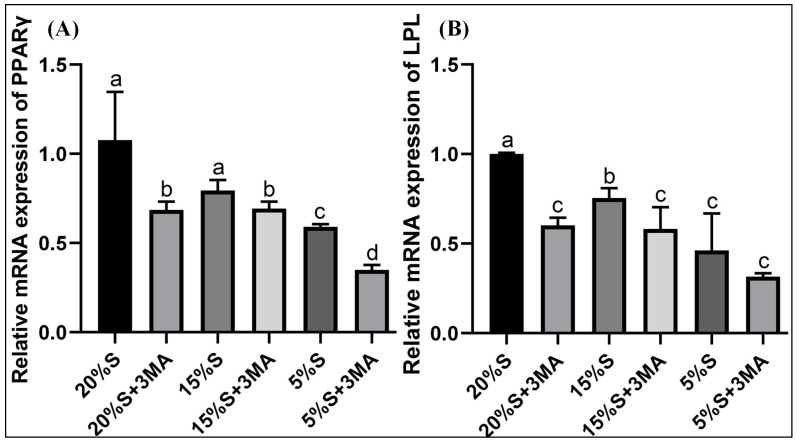
Detection and analysis of PPARγ and LPL by qRT-PCR. (**A**) Expression of PPARγ mRNA; (**B**) expression of LPL mRNA. The data are expressed as the mean ± SD (*n* = 3) of three experiments. The statistical significance of differences between groups was analyzed using one-way ANOVA, the same letters indicate the difference is not significant, while different letters indicate the difference is significant (*p* < 0.05).

The relative mRNA expression of PPARγ in the six groups were 1.08 ± 0.27, 0.69 ± 0.05, 0.79 ± 0.06, 0.69 ± 0.04, 0.59 ± 0.02, and 0.35 ± 0.03, respectively. Those of the LPL protein in the six groups were 1.00 ± 0.01, 0.60 ± 0.04, 0.75 ± 0.05, 0.58 ± 0.12, 0.46 ± 0.21, and 0.32 ± 0.02, respectively, shown in Figure 6. The expression of the two mRNA levels decreased gradually in the 20% S, 15% S, and 5% S groups. The protein expressions were lower in groups with 3-MA than in the corresponding groups without 3-MA.

#### 3.3.3. Content of Fat 

**Figure 7:**
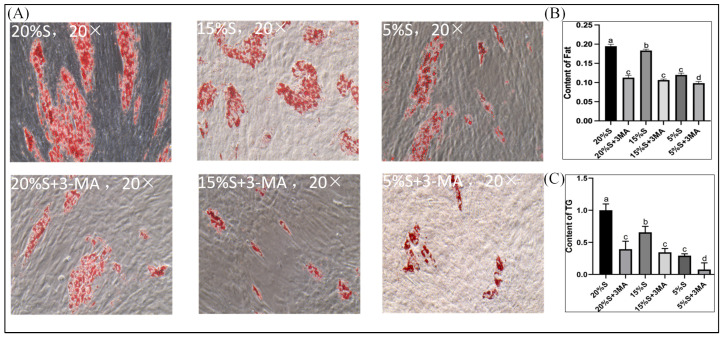
Detection and analysis of fat content and TG levels. (**A**) Detection of fat content (20×), the red part is stained fat, and the unstained surrounding is muscle; (**B**) quantitative determination of the OD value; (**C**) detection and analysis of TG levels. The data are expressed as the mean ± SD (*n*= 3) of three experiments. The statistical significance of differences between groups was analyzed using one-way ANOVA, the same letters indicate the difference is not significant, while different letters indicate the difference is significant (*p* < 0.05).

The cells in all groups contained orange-red lipid droplets, with the highest number and largest area of lipid droplets being observed in the 20% S group and the lowest number and smallest area being found in the 5% S group. The addition of 3-MA significantly reduced the number and area of lipid droplets across all groups, shown in Figure 7A. The optical density at 510 nm was measured using an Oil Red O staining kit, and the values for the six groups were 0.19 ± 0.01, 0.12 ± 0.01, 0.18 ± 0.01, 0.11 ± 0.01, 0.12 ± 0.01, and 0.09 ± 0.01, respectively, shown in Figure 7B. The values decreased gradually in the 20% S, 15% S, and 5% S groups (*p* < 0.01). The values were lower in groups with 3-MA than in the corresponding groups without 3-MA (*p* < 0.01). These findings were consistent with those obtained upon microscopic observation following Oil Red O staining.

#### 3.3.4. Content of TG 

The TG contents in the six groups were 1.00 ± 0.10, 0.39 ± 0.10, 0.66 ± 0.09, 0.35 ± 0.06, 0.29 ± 0.03, and 0.14 ± 0.01, respectively, shown in Figure 7C. The contents decreased gradually in the 20% S, 15% S, and 5% S groups (*p* < 0.01). The contents were lower in groups with 3-MA than in the corresponding groups without 3-MA (*p* < 0.05 or *p* < 0.01).

The results of Section 3.3.2, Section 3.3.3 and Section 3.3.4 indicate that serum starvation reduced adipogenic differentiation and that this reduction was positively correlated with the level of serum starvation. Additionally, autophagy played a role in alleviating the decline in adipogenic differentiation during serum starvation.

Overall, the results of Section 3.3 indicate that serum starvation ultimately led to the inhibition of SMSC differentiation. However, the results obtained from the autophagy inhibition groups indicate that autophagy significantly promoted SMSC differentiation during long-term serum starvation.

## 4. Discussion

SMSCs are undifferentiated myogenic precursors in adult muscle tissue that undergo myogenic differentiation to form muscle fibers when stimulated by specific signals [1]. We previously found that short-term mild serum starvation (15% S for 24 h) promoted SMSCs’ metabolism and enhanced their myogenic and adipogenic differentiation through the autophagic pathway [1,2]. These findings underscore the potential for the application of serum starvation to promote the growth of animal skeletal muscle. To better understand the impact of serum starvation on skeletal muscle growth, SMSCs were subjected to different levels of serum starvation simulated by reducing the serum concentration in culture media over an extended period (96 h). This approach allowed the investigation of the effects of serum starvation on SMSC metabolism, autophagy, and differentiation.

We measured the apoptosis rate, ROS levels, MMP, and ATP levels [14,15,16,17], which are commonly used to reflect cellular metabolic levels, to comprehensively evaluate the effects of long-term serum starvation on cell metabolism. We found that long-term serum starvation accelerated cell metabolism and apoptosis, negatively impacting cell growth, but that cells compensated for the energy deficiency by increasing ATP production and activating autophagy to mitigate these adverse effects. In contrast, our previous studies showed that short-term mild serum starvation increased ATP production in cells, while the apoptosis rate, ROS, and MMP were not significantly different from those in normal cells, thus promoting cell metabolism without affecting cell growth [10]. However, long-term serum starvation (mild and severe) showed inhibitory effects on cell growth and metabolism.

Starvation is a well-known stressor that triggers autophagy, and the quantity of autolysosomes and autophagosomes is commonly used to detect the extent of autophagy in cells [18,19]. Additionally, the autophagy marker proteins p62 and LC3B are frequently employed to measure the levels of autophagy [20,21,22]. The AMPK/mTOR signaling pathway is a well-established mechanism for autophagy, and the activation of AMPK and the inhibition of mTOR collaboratively initiate the autophagy process [23,24,25]. By measuring these indicators, we observed that long-term serum starvation induced autophagy via the AMPK/mTOR pathway, with the level of autophagy showing a positive correlation with the severity of serum starvation. This finding aligns with the previously observed effects of short-term serum starvation on autophagy in SMSCs [11]. Since multiple signaling pathways can cause autophagy, other molecular pathways that may modulate autophagy and muscle differentiation in response to serum starvation need further verification.

Low concentrations of horse serum in the culture medium have been shown to induce the differentiation of SMSCs into myocytes [26], while the medium used for induction of adipogenic differentiation promoted their differentiation into adipocytes [27,28,29,30]. The genes encoding MyoD1 and MHC are used as markers for myogenic differentiation [31,32], while PPAR*γ* and lipoprotein lipase are the primary transcription factors regulating adipogenesis [33,34]. Measurement of these indicators enables evaluation of the impact of long-term serum starvation on SMSCs’ differentiation at the molecular level. Our findings indicated that long-term serum starvation significantly reduced both myogenic and adipogenic differentiation of SMSCs, and that these changes were accompanied by notable decreases in lipid and triglyceride levels following adipogenic differentiation. Additionally, the extent of this reduction was positively correlated with the level of serum starvation. The results obtained from the autophagy inhibition groups indicated that autophagy significantly promoted SMSC differentiation during long-term serum starvation. However, the adverse effects of excessive ROS accumulation, reduced mitochondrial membrane potential, and increased apoptosis rates on SMSC differentiation during long-term serum starvation appeared to be more pronounced, ultimately leading to the inhibition of SMSC differentiation. Our previous research showed that short-term mild serum starvation (15% S for 24 h) promoted SMSCs’ differentiation, while short-term severe serum starvation (5% S for 24 h) inhibited their differentiation [10].

Based on the results of this experiment and our previous findings, short-term mild serum starvation significantly enhances the metabolism and differentiation of SMSCs. While autophagy induced by long-term serum starvation moderately promoted myogenic and adipogenic differentiation of SMSCs, this effect was insufficient to counteract the overall inhibitory impact of long-term serum starvation on cell differentiation. Therefore, we are currently exploring strategies to promote muscle development through controlled starvation. Specifically, we are using an intermittent mild fasting mouse model to investigate the effects of starvation on skeletal muscle growth. The results of mouse experiments indicate that intermittent mild fasting induced autophagy via the AMPK/mTOR signaling pathway. Compared with mice on a standard feeding protocol, the fasting regimen led to an increase in skeletal muscle mass and a decrease in visceral fat content, despite a reduction in food intake (data to be published).

## Data Availability

The datasets used during the current study are available from the corresponding author on reasonable request.

## Supplementary Materials

The following supporting information can be downloaded at: https://www.mdpi.com/article/10.3390/vetsci12010011/s1, File S1: The Original WB Figures.
