# Supplementary figures and images for: Effects of Long-Term Serum Starvation on Autophagy, Metabolism, and Differentiation of Porcine Skeletal Muscle Satellite Cells
Source: Vet Sci. 2024 Dec 30;12(1):11. doi: 10.3390/vetsci12010011 (PMC11768654; doi:10.3390/vetsci12010011)

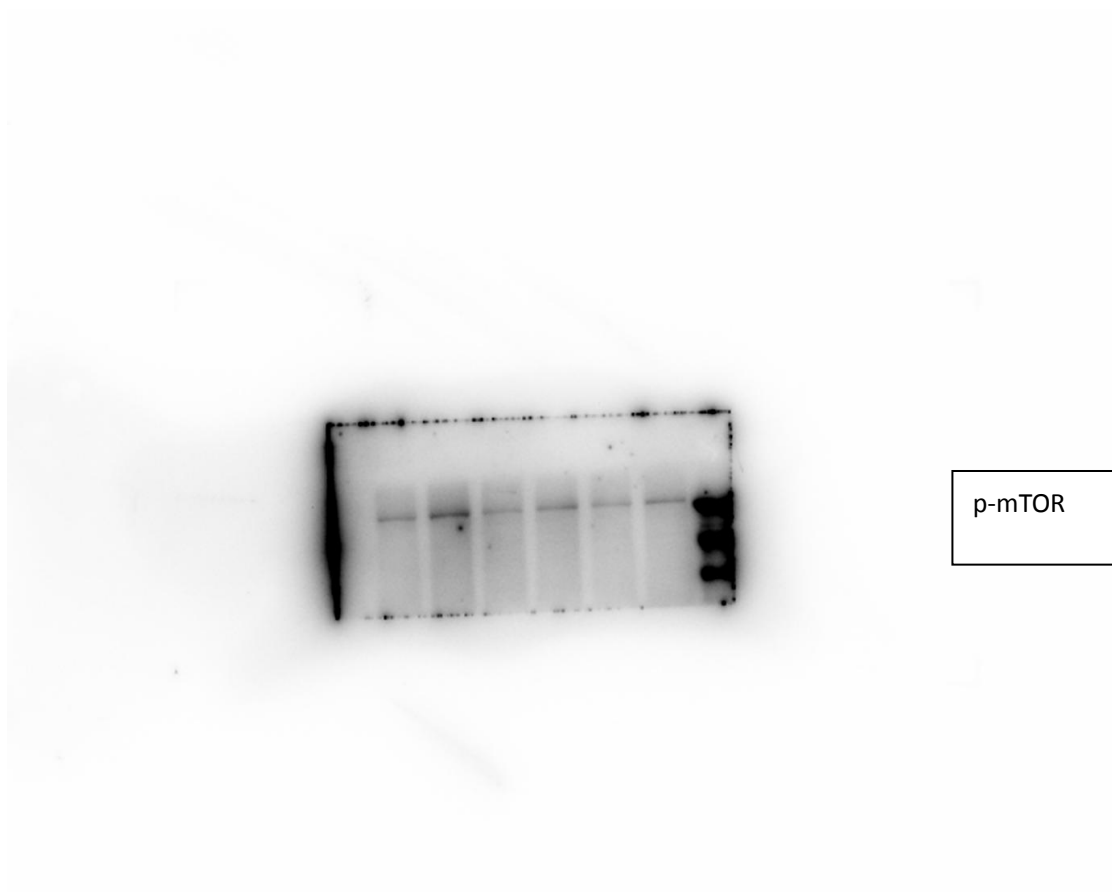

p-mTOR

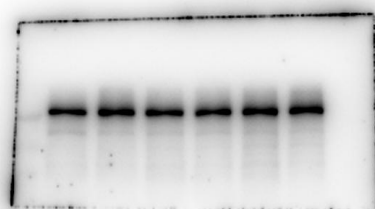

mTOR

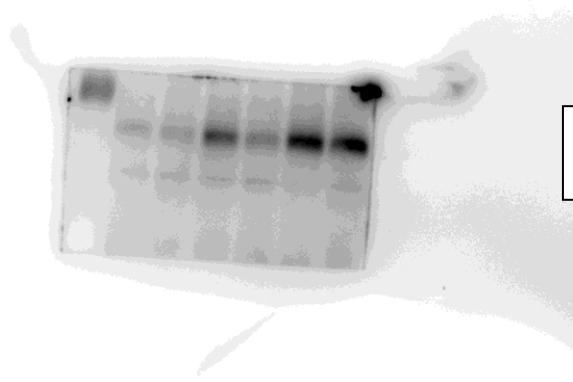

p-AMPK

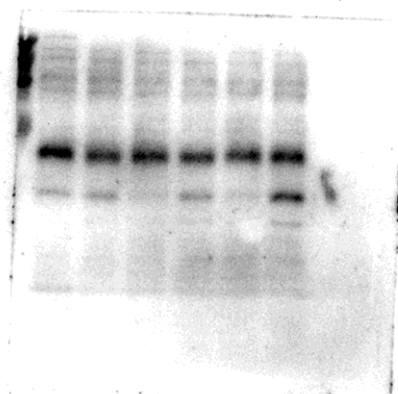

AMPK

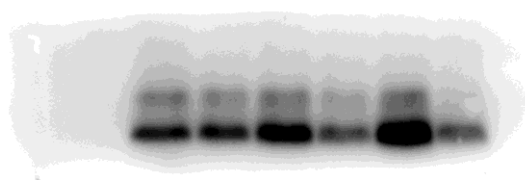

LC3

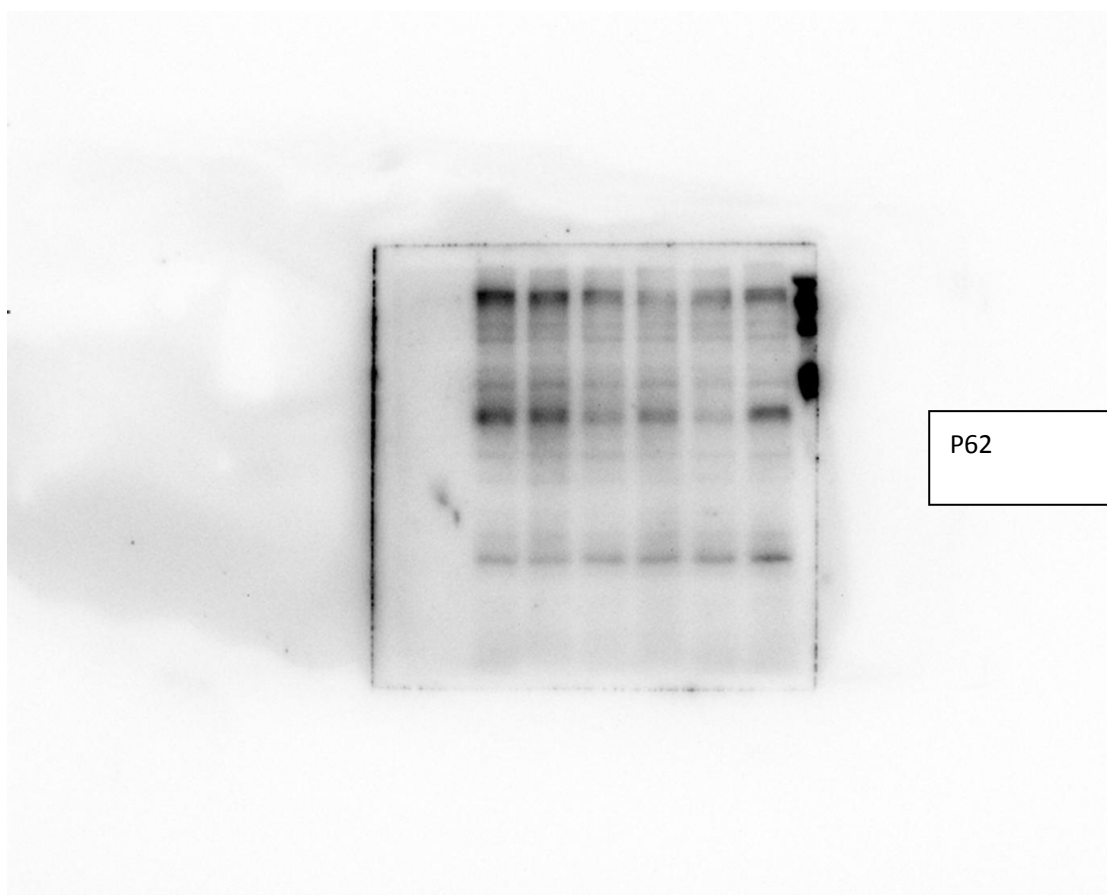

P62

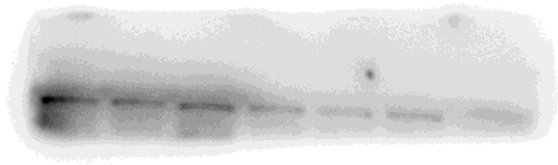

MHC

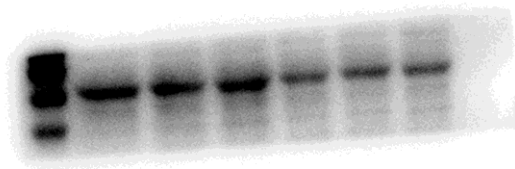

MyoD1

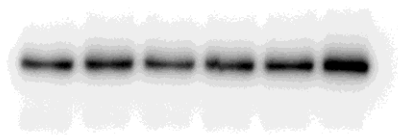

α-tubulin1

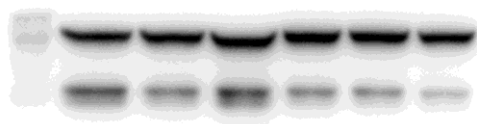

α-tubulin2

Supplement: Supplementary file 1 [file vetsci-12-00011-s001.zip › vetsci-3325931-supplementary.pdf]
